# Hard-to-Treat Areas in Psoriasis: An Underevaluated Part of the Disease

**DOI:** 10.3390/life15030425

**Published:** 2025-03-07

**Authors:** Adina-Mihaela Lupulescu, Alexandra Petruța Savu, Ştefana Bucur, Elena-Daniela Şerban, Sanda Popescu, Maria Magdalena Constantin

**Affiliations:** 1 Department of Dermatology, “Carol Davila” University of Medicine and Pharmacy”, 020021 Bucharest, Romania; stefana.bucur@umfcd.ro (Ş.B.); elena-daniela.serban@drd.umfcd.ro (E.-D.Ş.); maria.constantin@umfcd.ro (M.M.C.); 2 2nd Department of Dermatology, Colentina Clinical Hospital, 020125 Bucharest, Romania; 3 Department of Dermatology, Wrightington, Wigan and Leigh NHS Teaching Hospitals, Manchester M41 5SL, UK; sanda_escu@yahoo.com

**Keywords:** psoriasis, hard-to-treat areas, prevalence, quality of life, scalp psoriasis, nail psoriasis, genital psoriasis, palms and soles psoriasis

## Abstract

Hard-to-treat areas in psoriasis vulgaris, i.e., the scalp, nails, genital area, palms, and soles, are less commonly diagnosed and treated. Our understanding of the complex etiopathogenesis and treatment of psoriasis vulgaris has advanced considerably in recent years. After performing an English literature search, the present article is a comprehensive review based on several relevant articles. The articles included met the following criteria: they mentioned the “hard-to-treat areas, special sites, difficult-to-treat areas” or the specific body location of psoriasis, and they reported the psoriasis prevalence and/or patients’ quality of life. Despite the extensive information about psoriasis, there are still many limitations and challenges regarding the appropriate approach to psoriasis in these special locations. But emerging directions such as precise severity scores, new biomarkers for disease monitoring, and treatment decisions or forthcoming therapies represent solutions to improve the lives of those affected. Although they affect a small area, the impact on the quality of everyday life is significant, causing physical and mental disability. In this review, we try to highlight the need for more information about hard-to-treat areas, including their prevalence, a more rapid diagnosis, and a correct classification based on their real severity and their specific treatment before a significant impact on patients’ quality of life occurs. By presenting these challenges, we hope to contribute to efforts at improving disease control.

## 1. Introduction

Psoriasis is a chronic inflammatory immune-mediated condition that affects over 60 million people of the world’s population [1,2]. The elbows, knees, lumbar region are the most frequently affected areas of the body. Less common, lesions occur on the scalp (45–56%), nails (23–27%), genital (30–40%), palms and soles (12–16%), or in intertriginous regions (21–30%) [3,4].

**Figure 1:**
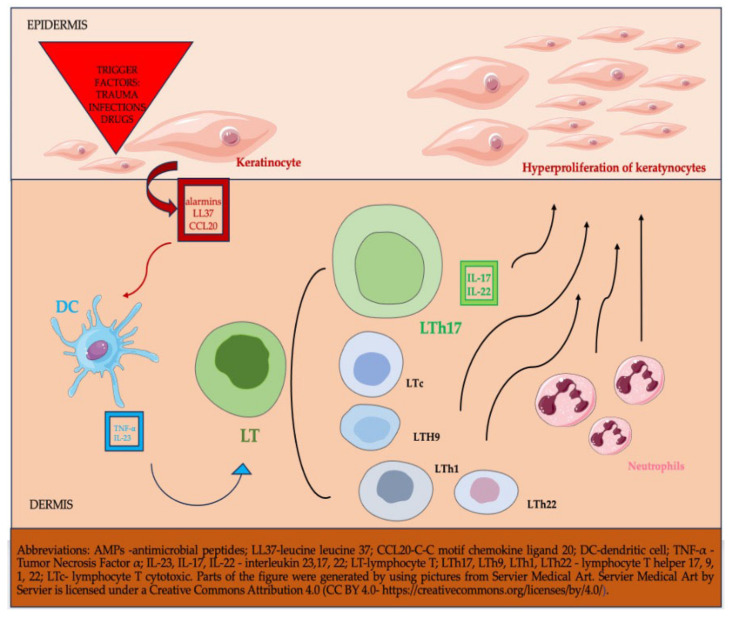
Basic etiopathogenesis in psoriasis.

Important findings about psoriasis etiopathogenesis have been revealed in the last decades. So far, we know that multiple factors play major roles: environmental risk factors (e.g., infections, trauma, drugs), multiple inherited alleles, and immunologic factors [5,6]. Immunologically, psoriasis is the result of skin inflammation due to the uncontrolled functioning of both the innate and adaptative cutaneous immune systems. Although the clinical manifestations are the result of a complex interaction between multiple cells, cytokines, and chemokines, we can state that its major role is that of lymphocyte T helper (LTh) 17 cells, dendritic cells, and keratinocytic cells. Dendritic cell release Tumor Necrosis Factor (TNF)-α and interleukin (IL)-23, which determines lymphocyte T (LT) differentiation in LTh 17, which release IL-17 and IL-22. The interaction of these molecules leads to activation and hyperproliferation of keratinocytes and skin inflammation. Other cells involved in this complex mechanism of psoriasis’ pathogenesis are represented by neutrophils, cytotoxic cells, Th9, Th22, Th1 with all the cytokines and chemokines they secrete (Figure 1) [7,8,9].

Psoriasis became an important global health problem, especially for its increasing prevalence [6]. Still, worldwide overarching epidemiologic data of psoriasis are lacking because the available data come from a limited number of countries. Thus, there is still much geographic data to be searched, as evidenced by a systematic review of the worldwide epidemiology of psoriasis in 2017 [10]. According to different articles, various disease prevalences are declared. Worldwide, the prevalence of psoriasis in adults is estimated to be between 0.51 and 11.43% [10]. To give an example, in Romania there are almost 400,000 people with psoriasis, and the prevalence of psoriasis vulgaris, according to a recently published study in 2021, was 4.99% and in a previous study 5.18%. As we know so far, in Romania there are only two epidemiological studies for psoriasis vulgaris, in 2019 and in 2021 [11,12]. Another article mentioned that the prevalence varies from 0.14% in East Asia to 1.99% in Australasia, with higher prevalence in Western Europe (1.92%, 1.07% to 3.46%), Central Europe (1.83%), and North America (1.50%). In adults, the incidence of psoriasis varies from 30.3 per 100,000 persons in Taiwan to 321.0 per 100,000 persons in Italy [13].

Regarding the worldwide prevalence of scalp, nails, genital, palm, and sole psoriasis, the information is incomplete, and new clinical trials must be conducted. Also, despite the considerable amount of information on psoriasis found in the literature, data about proper management and the disease’s impact on the quality of life (QoL) for patients with difficult-to-treat areas are limited [11].

Psoriasis vulgaris involving the scalp, nails, genital area, palms and soles can exhibit various features and may have a weaker response to commonly used treatments for psoriasis. Therefore, in recent years “special sites”, “hard-to-treat psoriasis”, “difficult-to-treat psoriasis”, and “challenging-areas” have been used as specific terms when we refer to psoriasis vulgaris localized in these areas [11,12].

Most patients with psoriasis have experienced social stigma, discrimination, and had an undesirable QoL at least once during their lifetime [10]. The impact on QoL is also, frequently, linked to the occurrence of lesions in special anatomical sites such as the scalp, nails, genital area, or palms and soles, which are often associated with functional impairment [3].

The objective of this review based on extended literature research was to collect data about psoriasis in hard-to-treat areas, in particular the prevalence of these hard-to-treat areas, effective treatment options, and the impact on QoL. After performing the research, we found insufficient data on this topic, so we needed to focus carefully only on this specific type of psoriasis in our daily clinical practice, collect new data, and publish them. This type of article may encourage physicians to concentrate more on these sensitive zones by possibly increasing prevalence, influencing treatment decisions, and decreasing the psoriasis burden in all its aspects: treatment ineffectiveness, impaired QoL, economic burden.

## 2. Materials and Methods

The following review is based on a broad literature search of over 100 screened references. Only 62 relevant articles to the current problem met the research criteria. We have used keywords as “psoriasis”, “hard-to-treat areas”, “psoriasis in special sites”, difficult-to-treat areas”, “scalp psoriasis”, “nail psoriasis”, “genital psoriasis”, “palms and soles psoriasis”, “psoriasis prevalence”, “quality of life in psoriasis”, and we performed searches in the databases PubMed, Web of Science, and SpringerLink. Articles included met the criteria by containing the statements “hard-to-treat areas/special sites/difficult-to-treat areas”, or each specific location of psoriasis, reports about the psoriasis prevalence and patients’ quality of life, combined in the same article or individually. We generated the current work after discarding materials published before 2013 and in other languages than English.

## 3. Hard-to-Treat Areas: Current Challenges

*a*. *Prevalence of psoriasis in hard-to-treat areas—few known data, a lot to be searched.*

Psoriasis vulgaris’s incidence and prevalence vary worldwide due to multiple factors and are incompletely known [1,13,14]. Epidemiological data are even less determined when we talk about psoriasis in hard-to-treat areas, as was already mentioned above.

Finding out data about the disease’s prevalence and incidence brings an important benefit for worldwide physicians to focus more carefully on the disease and particularly on difficult-to-treat areas in psoriasis. Also, by knowing precise data, information campaigns can be made, both for the patients to acquire knowledge of their condition, with its risk factors and appropriate guidance, but also for the allocation of necessary funds for the proper management of the disease [15].

Geography, ethnicity, environment, and genetics could be major factors in the variation of the prevalence of psoriasis vulgaris [14]. Also, the prevalence may be influenced by the severity of the disease or by its diversity in affected areas. For example, Caucasians compared with other human races have higher prevalence rates [14]. Within Europe, the prevalence seemed to be higher in countries like Italy, France, and Norway compared with the UK [14]. In comparison with other European countries, psoriasis in Romania seems also to be distributed differently across the country, and this could be explained by environmental factors, financial conditions, different types of stress, or even genetic features [11].

Psoriasis prevalence among adults ranges from 0.4% in Asian countries to 8.5% in Norway [14] and is estimated worldwide to be between 0.51% and 11.43% [10] to date. Prevalence data only on the hard-to-treat areas in psoriasis are limited, with isolated studies on this topic. For example, in a recent article about nail psoriasis among Malaysian patients with psoriasis, the stated nail prevalence was 54.2% [16]. In many cases, psoriasis in special areas is not considered severe or extended enough to be included in epidemiological studies [11,12].

*b*. *Diagnosis, severity, and treatment of psoriasis in hard-to-treat areas.*

Hard-to-treat locations in psoriasis are frequently the only clinical presentation of the disease, and even in severe forms their recognition is sometimes delayed, making the correct and early diagnosis challenging [3,17]. After the correct diagnosis has been confirmed, the severity of the disease must be established in order to have the appropriate approach of the condition.

Using assessments reported by both the physician and the patient for a comprehensive definition, psoriasis severity is classified differently worldwide. Although there are many systems to evaluate the severity, none of them are accurate enough to make a clear division between mild, moderate, and severe forms of the disease in order to determine the best treatment. In daily practice, different tools like the Psoriasis Area and Severity Index (PASI), Dermatology Life Quality Index (DLQI), Body Surface Area (BSA), and the Physician’s Global Assessment (PGA) are used to create a precise severity scoring system [17,18,19]. PASI, one of the most commonly used scores in clinical practice, includes erythema, thickness, scale, and the percentage of affected area, to evaluate psoriasis severity and its response to treatment. But the score has several limitations that can reduce its practical precision and usefulness. For example, it does not include the special sites of psoriasis, and the percentage of the affected areas can vary depending on who is estimating them [17]. The situation is similar for BSA or PGA severity classifications. Thus, it is even more difficult to have accurate knowledge when it comes to hard-to-treat areas and their appropriate severity classifications [18,20].

**Figure 2:**
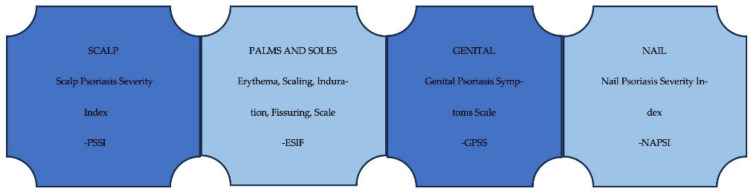
Severity scores for hard-to-treat areas in psoriasis.

For special areas, different specific scores are used: Scalp Psoriasis Severity Index (PSSI), Nail Psoriasis Severity Index (NAPSI), Erythema, Scaling, Induration, Fissuring Scale (ESIF), Genital Psoriasis Symptoms Scale (GPSS), but none of them is included in the standard scores mentioned above (Figure 2) [4,17]. Also, a score was developed to measure the general psoriasis severity that includes patient-reported outcomes equally weighted with physician-assessed disease activity index scores: Brigham Scalp Nail Inverse Palmoplantar Psoriasis Composite Index [4,21,22].

In a recent article, two options are proposed for evaluating psoriasis severity in special sites: either calculating the highest score for that specific area or modifying the PASI score with a correction factor for each difficult-to-treat area [17]. For example, in Romania we consider a severe form of the disease if severity scores for each area are above a certain threshold value. Precisely, NAPSI ≥ 32, PSSI ≥ 24, ESIF ≥16, PGA ≥ 4 define a severe psoriasis in a special area and guide treatment decisions [17,23]. From another recent article, we found out that the International Psoriasis Council (IPC) proposed a new, more targeted and specific method of classifying psoriasis severity considering sensitive areas involvement, treatment history, and BSA. Thus, disease severity should be classified by using both this new method that includes supplementary details and classical measures. IPC recommendations for systemic therapy in psoriasis are: patients with special areas involved, BSA > 10% and failure of topical therapy [18,22]. This new method tries to integrate difficult to treat areas in severity classification systems for proper diagnosis and treatment [18].

In order to decide the best management plan, in addition to the clinical severity classification, physicians should bear in mind that psoriasis has great clinical heterogeneity, different treatment responses, and different disease durations and evolutions. Also, other possible comorbidities that the patient may have, and the patient’s lifestyle, should be considered [3,4,17]. Psoriasis could be associated with several comorbidities including metabolic syndrome with all its components: obesity, diabetes melitus, dyslipidemia, hypertension and cardiovascular diseases or psychiatric diseases, and inflammatory bowel diseases [24]. Also, factors such as smoking, alcohol consumption, daily stress, sedentarism, and unbalanced diets can negatively impact psoriasis’s clinical presentation, evolution, treatment response, as well as other comorbidities and the QoL. Current knowledge indicates that avoiding smoking, alcohol consumption, and acute psycho-emotional stress, or doing regular physical activity, can prevent psoriasis exacerbations. Also, diets with low caloric intake and high consumption of antioxidants (fruits, vegetables) can be useful alongside conventional therapies. Promising results were obtained especially regarding diet interventions and stress reduction, but the studies carried out up to this point have not been able to state with certainty a direct link between these lifestyle or diet changes and the improvement in the severity of the disease and the QoL or the proportion of the two influences each other. Therefore, new studies are needed to associate interventions on lifestyle and diet to the QoL of patients with psoriasis [25,26].

The main treatments currently available for psoriasis in hard-to-treat areas consist of topical therapies, systemic and targeted phototherapy, systemic agents such as cyclosporine, methotrexate, or apremilast and biological therapies [4,27,28]. However, there is still no consensus on which is the best treatment option for special sites. In daily clinical practice, there are cases where topical therapies are ineffective [17], so the management of psoriasis in such localizations often needs systemic conventional or biological therapies [17,29]. Recently developed selective biological agents such as adalimumab, brodalumab, bimekizumab, certolizumab, etanercept, infliximab, ixekizumab, golimumab, guselkumab, secukinumab, risankizumab, tildrakizumab, and ustekinumab, which are directed against different important cytokines (TNF-α, IL17 family members (IL17A, IL17C, IL17F), IL-23 and IL12/IL23) by blocking the inflammatory cascade at different levels, are widely used for moderate-to-severe forms of psoriasis [30]. However, in milder forms of the disease but with difficult-to-treat area involvement or joint involvement, biologic drugs may be required. Still, if their efficacy is proven, their use in special localizations or in small body surface areas of psoriasis remains limited, especially because this type of medication needs to comply with certain criteria to be prescribed [23,28,31]. Ixekizumab is one of the biological medications approved for psoriasis, and recent studies have determined its meaningful effectiveness in psoriasis in difficult-to-treat areas [31]. Also, in addition to ixekizumab, which is effective especially for genital psoriasis, other promising options to treat these difficult-to-treat areas are sekukimumab and risankizumab, with high efficiency in disease control, sekukimumab being effective for psoriasis involving palms and soles and risankizumab in patients with scalp and genital psoriasis [31,32]. The arrival of these new agents holds promise for better control of the disease of those affected. But we must not forget that, even when the right treatment is chosen, and its effect is long-lasting, there are cases with disease recurrence in the same sites after therapy withdrawal [33]. From daily practice, we can state that hard-to-treat areas may also be prone to recurrence.

To choose the best treatment option for a challenging-to-treat area, doctors must consider the clinical characteristics of that area, if there is local impairment, disease activity, and patients’ expectations, as these may influence the therapeutic result [27]. As evidenced by a Portuguese study published in 2023, an important percentage of patients with lesions in difficult-to-treat areas need systemic therapy as soon as the diagnosis is made, but they are undertreated since the low efficacy of conventional therapies leads to lower compliance and aggravates the control of the disease [34].

Recently, serologic evaluations with specific inflammatory biomarkers such as vascular endothelial growth factor, high-sensitivity C-reactive protein, metalloproteinase-3 or dermoscopy have been proposed for use in daily practice to assess clinical severity and to measure treatment response, alongside more commonly used specific severity scores [30]. It remains to be seen if these evaluations could also be useful for sensitive areas.

The main purpose of treating patients with psoriasis in difficult-to-treat areas remains to clear the affected area and to reduce the impact on QoL [27]. Very few controlled trials make correlations between the severity of psoriasis localized in hard-to-treat areas, the efficacy of the therapy prescribed, and the impact on the QoL. The data we have in general are obtained from studies involving patients with psoriasis lesions in common sites, who also have involvement of these special sites [28].

*c*. *The impact of psoriasis, including hard-to-treat areas, on patient’s quality of life.*

Psoriasis vulgaris is an important cause of social, psychological, and physical disability with a major impact on patients’ QoL. Quality of life is the most frequently patient-reported result in medical practice and can be defined in several complex ways, but when we refer to good health, first, we must take into consideration the patient’s physical and psychological status [35,36]. Even physical changes in different body areas, such as sensitive areas, are the ones that impact the patient’s life first, though perhaps psychological burden is the one that contributes most to the decline on QoL, patients frequently facing a loss of confidence, the perception of being stigmatized, anxiety, and depression. There is an interdependence between a patient’s mental status and his clinical manifestation with a negative effect on QoL [37,38]. Very often patients with psoriasis, including special sites, experience unmanageable clinical manifestations of the disease, social isolation, difficult interpersonal relationships, or even fear of transmitting their disease to their children [14,34,39].

There are several different factors associated with greater impairment of QoL in psoriasis: disease severity, disease localization in isolated areas (scalp, nail, genital, palms, soles), younger age, lower educational levels, lower incomes, urban settlements, higher PASI scores, and inadequate treatment [37]. There is also a greater association between the substantial reduction in QoL and anxiety and depression symptoms in women or in patients who have associated comorbidities (such as psoriatic arthritis), as evidenced by DLQI or PDI [37]. Also, the disease has a ‘relapsing and remitting’ course that adds a supplementary burden [36], and thus the patient’s awareness of psoriasis’s severity is different.

The localization of psoriasis in sensitive areas, especially the severe forms, often impairs the normal function of the affected area [3], but the degree of dysfunctionality can be underestimated due to the reduced body surface of these special sites. Often, when the disease is not considered severe enough to start therapy or there is a delay in the appropriate therapy, this can lead to a prolonged disease course and a negative impact on QoL in the long term. Also, cases recalcitrant to treatment greatly impact QoL [4,17,27].

Psoriasis is a chronic disease which needs long-term treatment that can be a significant burden in patients’ everyday life. Patients must be aware of, accept, and understand the long-term use of treatments and learn how to manage the impact of these treatments on their physical and behavioral activities and their psychological or everyday workload. Together with their patients, health practitioners must conceive the proper treatment regimen to obtain the best compliance, maximize therapeutic effects, minimize treatment-related side effects, make the burden easier to bear, and reduce the impact of the disease on the patients’ lives [39].

A variety of tools to assess QoL in patients with psoriasis have started to be used in daily practice. Among these are disease-specific and dermatology-specific QoL tools like DLQI, Short-Form 36-Item Health survey (SF-36), and the Psoriasis-Disability Index (PDI) [37]. Among the simplest instruments is PDI, which contains several questions relevant to a patient’s perception [38], but DLQI is the most commonly used tool in daily practice. Recent studies reported that psoriasis in special areas correlates with higher DLQI scores, worse QoL, and higher rates of depression [40,41]. However, a recent article that analyzed the link between happiness, QoL, and DLQI stated that DLQI is not accurate enough to measure the psycho-emotional burden as a whole because its main purpose is not to measure the patient’s emotional status, but rather to measure the physical dysfunction that also associates with negative discomfort [35]. Also, in addition to the emotional aspect, DLQI fails to adequately capture the mental aspects of the patient’s QoL and has limitations when it comes to measuring coping strategies, therapies and side effects, long-term disease, and certain limitations in job options [42]. None of the previously mentioned tools are adapted for hard-to-treat areas, often the measurements being carried out in patients with plaque psoriasis who have also sensitive areas involved.

From a very recent review article, future directions show a new attempt to identify new biomarkers associated with psoriasis activity, therapy response, and QoL [37]. These new directions can be promising, but it will take time to adapt them for special areas and include them in daily practice.

Therefore, we need better information about the population with psoriasis in special sites, with their different characteristics and specific treatment options, in order to improve patient QoL [34].

### 3.1. Scalp Psoriasis

Scalp psoriasis is a common, but at the same time a special expression of the disease. It can be the only manifestation of psoriasis, or it can be associated with lesions in some other areas of the body and is included in the “hard-to-treat” manifestations of psoriasis [43].

The scalp is among the first and the most frequently special-affected areas in psoriasis, occurring in almost 45–56% of patients with psoriasis [4].

**Figure 3:**
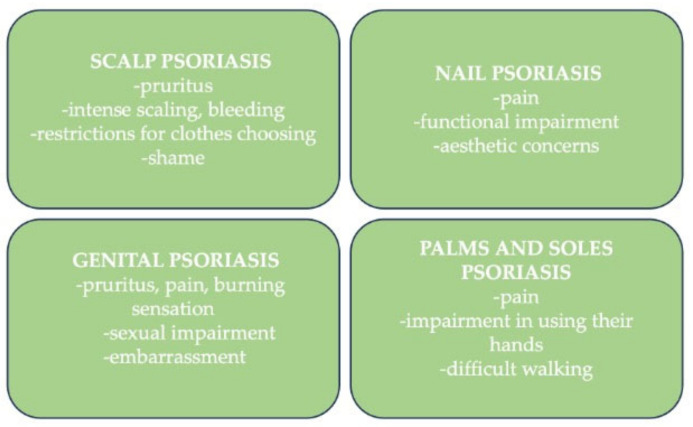
The daily burden of patients affected by psoriasis in hard-to-treat areas.

**Figure 4:**
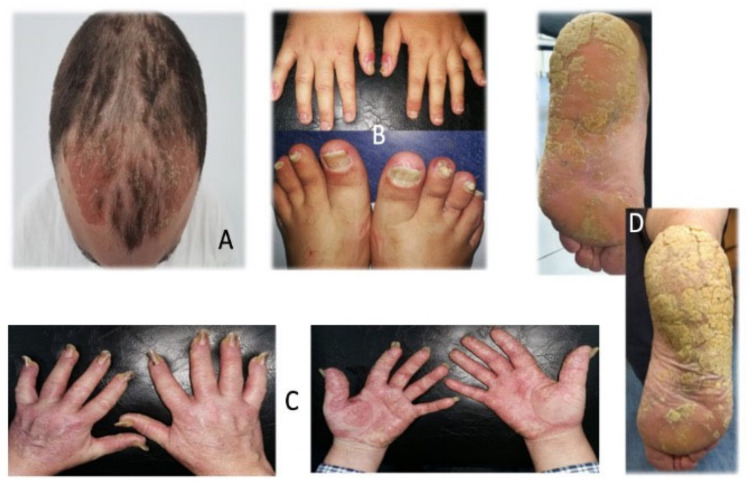
Hard to-treat areas in psoriasis. (**A**) Scalp psoriasis (courtesy of Dr. MM Constantin). (**B**) Nail psoriasis (courtesy of Dr. MM Constantin). (**C**) Nail and palms psoriasis (courtesy of Dr. MM Constantin). (**D**) Plantar psoriasis (courtesy of Dr. MM Constantin).

The main symptom is pruritus, which can be intense and disturbing. Along with severe scaling, pain, clothing restrictions, and feelings of embarrassment can have a significant psychological impact on psoriatic patients (Figure 3 and Figure 4A) [4,44].

It is also important to mention that localization of psoriasis in this area can be a prognostic factor for developing psoriatic arthritis [43].

The specific, most widely used instrument to evaluate and keep under observation scalp psoriasis is PSSI. The score evaluates scalp erythema, infiltration, and desquamation, establishing the severity of the disease, and is useful to guide treatment options and efficacy [17].

Topical corticosteroids and topical vitamin D analogs are among the most common treatment options for patients with scalp psoriasis. These topical agents can be enough for the initial stages, but with disease duration and progression treatment can be hard to use and sometimes unacceptable for many patients because of the difficult application due to the hair. This can lead to dissatisfaction, nonadherence to treatment, and impaired QoL. Thus, use of systemic therapy is much needed for these patients with hard-to-treat psoriasis. Recent review articles about scalp psoriasis and biological therapy showed that biologic therapies used for skin plaque psoriasis are also efficient in scalp psoriasis [43,45]. Of all recently developed biologics, ixekizumab, guselkumab, infliximab, brodalumab, and risankizumab appear to have the highest effectiveness [32,45].

Even in cases when biological therapy properly controls the disease, localization of psoriasis on the scalp remains a challenge because treatment may take longer to be effective and clear the skin. There are also cases where the scalp has no visible lesions and the treatment is considered efficient, but symptoms like pruritus or discrete scaling can persist and still be considered a burden for the patients [4,43,44,45,46].

DLQI, as a tool to measure QoL for psoriatic patients, includes scalp pruritus and scalp scaling assessment, but does not offer detailed information about the impact these mild remaining manifestations could continue to have, even for patients under efficient treatment and with low DLQI scores. Thus, physicians should evaluate the efficacy of the treatment after detailed discussion about the disease’s impact on the patient’s daily life [43].

Despite being a small affected area, the physical and psychological burden of psoriasis can be significant, remaining a great challenge for physicians. In order to enhance patients’ daily life, controlling scalp psoriasis in a thorough and efficient way is essential [45].

### 3.2. Nail Psoriasis

The nail is one of the most burdensome to manage sites of psoriasis, and in some cases, 5–10% according to a recent review, it can be the only manifestation of the disease without any cutaneous manifestation [47]. Nail psoriasis can affect the nail bed or nail matrix and can have different clinical forms. The main symptom is the pain which can be disturbing in everyday life and can lead to significant psychological impact. Also, patients can experience functional impairment and esthetic unease (Figure 3 and Figure 4B,C) [47].

Almost 23–27% of patients with psoriasis have nail impairment. Patients can have modified manual dexterity, limitations in daily activities, or routine and cosmetic and self-image anxiety, especially women [4,48].

Psoriasis in this difficult-to-treat area can precede psoriatic arthritis by many years, thus making it a negative prognostic factor. Recent studies have shown that patients with nail psoriasis have three times more chances to develop psoriatic arthritis because nail manifestations can be linked with subclinical enthesitis, which is often seen in early stages of psoriatic arthritis. Thus, an early and correct diagnosis leading to the rapid and accurate treatment of nail psoriasis may delay the onset of psoriatic arthritis [47,49,50].

According to recent articles about nail psoriasis, its prevalence varies from 6.4% to 81.8%, or was stated to be 54.2%, but it is hard to know the real prevalence of this specific site because the few existing prevalence studies were made in cutaneous psoriasis that also has nail involvement [16,50].

The most frequently used severity scores for nail psoriasis are NAPSI, with its versions: modified NAPSI, target NAPSI, and Psoriasis Nail Severity Score (PNSS) [47,51]. NAPSI is numerically helpful and the most-used instrument in daily practice to evaluate and monitor psoriasis, and which can influence the choice of the proper therapy [51,52]. Actually, all of these scores have their impediments in daily use because nail psoriasis has heterogenous clinical manifestations [51]. Considering its large clinical variability, there is not yet a generally accepted best treatment for nail psoriasis. Treatment choice is made by disease severity and extension ranging from topical to systemic treatments. According to a group of experts, recommendations for topical therapy are only in cases in which the disease is mild (≤3 nails involved) and for systemic therapy for multiple nails affected, resistant nail disease, or in patients with affected QoL [47]. The most frequently used topical therapies are glucorticoid and calcipotriol agents, topical tacrolimus, intralesional glucocorticoids, or, according to recent data, intralesional methotrexate [47]. However, topical therapies are not always suitable for psoriasis at this site because they do not penetrate across the nail plate. Also, intralesional agents may cause pain and may be refused by the patient. Thus, systemic therapy, conventional or biologic, may be necessary. New agents such as ixekizumab, secukinumab, ustekinumab, and apremilast are more suitable for nail psoriasis, showing that these patients can improve their symptoms and QoL sooner even if their disease cannot be classified as a severe form of disease according to the severity scores. However, their price and lack of indications for nail-limited psoriasis reduce the current use [4,47,50,51].

Nail psoriasis needs wide attention and is not yet included in large studies because it is often underrecognized. This leads to underevaluated and undertreated disease or underestimated severity and great stress on the patients’ QoL [51].

### 3.3. Genital Psoriasis

Genital psoriasis is the most stigmatizing form of the disease, but even so there is limited information and awareness about psoriasis involvement in this sensitive area. Its main manifestations, such as pruritus, pain, burning sensation, redness, and fissuring, frequently cause embarrassment, sexual dysfunction, or reduced sexual activity and depression with a major impact on QoL (Figure 3). This form of psoriasis affects about 63% of psoriasis patients [53,54,55,56].

Genital psoriasis seems to be more common in men [4]. Until recently, this form of psoriasis has been underestimated or was not treated with proper attention in the group of health professionals treating psoriasis, forgetting about the related psycho-sexual impact. Also, patients hesitate to discuss their lesions or symptoms with their physicians because they feel ashamed [54]. Few patients state that they had a previous examination of the genital area to determine if this site is affected, so the diagnosis is most often delayed, and the treatment initiated after a long period from the onset of the disease. So far, there are no specific frequently used tools to assess genital psoriasis severity or its response to treatment. A scale has been proposed which would measure the severity of many genital symptoms—the Genital Psoriasis Symptoms Scale (GPSS), but is not yet widely used in daily practice [57].

Included in the difficult-to-treat areas, psoriasis on this site seems to pose many challenges when it comes to proper treatment [58]. As in other sensitive areas, topical corticosteroids, and topical vitamin D analogs are the first treatment option for mild to moderate genital psoriasis. There are limited data about how long we can use topical corticosteroids without side effects and, also, about the long-term use of topical vitamin D analogs or other topical immunomodulators [4,58]. However, in severe cases of genital psoriasis, systemic treatment is needed. In recent years newer therapies have focused on key cytokines in psoriasis pathogenesis, in particular on IL- 23 and IL-17A. Drugs like ixekizumab, which targets IL-17A and is effective for genital psoriasis [4,31,58,59], have been developed. Research on the etiopathogenesis is progressing rapidly, so new molecules that target both IL-17F and IL-17A, such as bimekizumab, have been developed. Until now, even a recent study presented the important effectiveness of this agent in the treatment of genital psoriasis, though the study has several limitations and bimekizumab’s effects on the genital area has to be better studied regarding its improvements and long-term efficacy [58]. Another recently published study about apremilast, a new distinctive phosphodiesterase 4 inhibitor approved for psoriasis treatment, showed good results regarding genital psoriasis severity, signs, and symptoms, even in the long-term disease course [53]. However, until this moment there is no consensus about biological treatment in genital psoriasis and no available guidelines for this difficult-to-treat area. The few real-world experiences published to date reported that the only biological drug specifically accepted for genital psoriasis is ixekizumab, proving its important effectiveness with long-lasting results [31,58]. As in the case of the long-term use of topical therapies, the data for systemic therapies also remains limited [53].

According to a recent article about future trends in psoriasis, other encouraging upcoming therapies for psoriasis are new specific IL-23 inhibitors like mirikizumab, retinoid-associated orphan receptor γt (RORγt) inhibitors, or topical therapies used with microneedles and nanoparticle-based carriers, promising more targeted and personalized treatment plans [59]. This could be a useful solution for genital psoriasis and for other sensitive areas.

Patients affected by psoriasis in the genital area are more frequently prone to isolation, avoiding social activities or intimate relationships. Genital psoriasis impacts perhaps the worst of all the sensitive areas in the everyday life of affected patients [60].

### 3.4. Palms and Soles Psoriasis

Palms and soles psoriasis, often named palmo-plantar psoriasis, can only be manifested as an independent disease or may be associated with other localizations of psoriasis. Patients experience frequently intense pain and impairment in using their hands and walking (Figure 3 and Figure 4C,D). This difficult-to-treat area has the greatest functional disability and, also, is greatly stigmatized and rejected by people who do not know that psoriasis is not a contagious disease. Approximately 12–16% of psoriasis patients have palmo-plantar psoriasis [4].

In daily practice, the most-used score and a useful instrument to assess palms and soles severity is ESIF, but like other specific scores developed for hard-to-treat areas it has impediments when it comes to deciding the treatment and monitoring the disease evolution or impact on patients’ QoL. Even for severe forms, palms and soles affect < 5% of total body surface area, placing this impairment in the mild category of the disease according to the severity scores used in practice. Thus, its severity may be underevaluated and the proper treatment delayed [4,17,23,61].

As for other forms of difficult-to-treat psoriasis, topical and systemic therapies have been tried too, but these areas are sometimes characterized by resistance to topical and even systemic treatments. Among the most-used treatment options remain potent topical steroids, phototherapy for early stages, and, in more severe cases, systemic therapy such as acitretin, methotrexate, or biologic therapy [4,61,62]. There are cases where the application of topical treatments such as creams and ointments can limit some daily activities, making systemic therapy the preferred option even in the early stages. Secukimumab is one of the biological agents approved for psoriasis, and a recent study has determined its meaningful effectiveness in palmo-plantar psoriasis [32].

Palms and soles psoriasis has a major impact on QoL, with many patients suffering physical restrictions for certain activities, professions, or social life, significant psychological stress, and esthetic concerns due to these exposed areas. Even though it is an important component for long-term control of the disease, the QoL of patients with this difficult-to-treat area have rarely been studied independently, so further trials that focus only on this challenging area must be performed [61,62].

## 4. Discussion

Psoriasis in hard-to-treat areas impacts in multiple dimensions and significant proportions the everyday life of patients. Although only a small part of the body is affected, the patients’ daily burden should not be underestimated.

Even after a prolonged disease course it can be hard to get used to certain limitations of the daily routine due to the physical impairment that psoriasis in these areas can cause. Also, the disease can have a meaningful psychological impact, as patients may have limited understanding and acceptance of their condition. Thus, there is the associated risk of avoiding certain jobs, work abandonment, or frequent absenteeism and social isolation.

For a comprehensive management plan for psoriasis in challenging sites, specific diagnostic evaluation criteria and individualized therapies are required. Screening methods for early identification of the disease and prompt initiation of treatment are needed.

One of the biggest problems remains detecting the real severity in these special regions because in everyday practice severity scores for each area are not so often used when it comes to establishing a rapid diagnosis and determining treatment. However, the two above-mentioned options that have been proposed to evaluate psoriasis severity in special sites have not been measured in everyday medical practice. Even if systemic therapy is not always recommended because special areas represent a small proportion of the total body surface, when it is chosen for these specific areas, in severe cases, different individualized drug regimens and doses should be considered.

It is also important to focus not only on clinical measurements and treatment effectiveness but also on patient’s perception of his disease, his expectations, and his QoL. Physicians should be aware of counselling the patient when needed and measure in a proper way psychological signs like restricted communication or shame during medical consultations. Furthermore, there are no scores to measure the impact on QoL for challenging areas in psoriasis. The severity scores used daily like PASI or PGA cannot measure the impact on QoL in these special areas and the frequently used index DLQI should include specific criteria for measuring emotional manifestations with major impacts such as pain or pruritus and other particular aspects of each special site. It is necessary to develop new scores that connect the QoL with the real severity of the disease, and to guide the proper care required.

We must not forget that psoriasis is a complex disease, associated with different comorbidities, and request a multidisciplinary approach for the best decisions concerning the patient’s condition.

To sum up, there are not enough epidemiological and clinical data for psoriasis in these areas, so future studies that include only patients with these affected areas are needed. First, there is a real need to collect precise and first-rate epidemiological data only about special sites-psoriasis in order to have a better view of the distribution of the ailment. Secondly, a consensus must be reached on the definition of the specific severity scores and to be integrated into everyday medical practice. Also, it is essential to establish a diagnosis and a treatment algorithm based on the severity scores specific to each area, and to make a correlation between disease severity before and after initiation therapy, with the impact on patient’s QoL.

Although there is much information and even more in development in the scientific literature about psoriasis and hard-to-treat psoriasis, there is still a great deal of information to be completed for proper management of the disease.

This review tries to provide the first compiled and accessible data about hard-to-treat psoriasis, and to highlight the demand for further medical papers with new necessary additional information and new clinical guidelines, for a significant number of patients with decreased QoL due to this type of psoriasis. When psoriasis is incompletely managed, it becomes a real burden for the patients, for the doctors, and for the healthcare system.

Addressing special sites in psoriasis requires teamwork among researchers who communicate actual challenges and new discoveries, and clinicians who apply these new discoveries in everyday practice in order to solve actual current problems and reduce the decline of quality of life for the affected patients.

Although psoriasis is a chronic disease that has no curable treatment so far, it is possible to come up with a comprehensive healthcare strategy to manage the disease starting with an early diagnosis and proper treatment. The main focus must be directed at reducing physical and psychological impairment, recognizing and collaborating with different physicians to treat associated comorbidities, and implementing educational measures and changes in the patient’s lifestyle.
